# A Novel Bivalent Mannosylated Targeting Ligand Displayed on Nanoparticles Selectively Targets Anti-Inflammatory M2 Macrophages

**DOI:** 10.3390/pharmaceutics12030243

**Published:** 2020-03-08

**Authors:** Peiming Chen, Xiaoping Zhang, Alessandro Venosa, In Heon Lee, Daniel Myers, Jennifer A. Holloway, Robert K. Prud’homme, Dayuan Gao, Zoltan Szekely, Jeffery D. Laskin, Debra L. Laskin, Patrick J. Sinko

**Affiliations:** 1 Elucida Oncology, Inc., Monmouth Junction, NJ 08852, USA; pmchenru@gmail.com; 2 Department of Pharmaceutics, Ernest Mario School of Pharmacy, Rutgers, The State University of New Jersey, Piscataway, NJ 08854, USA; xzhang@pharmacy.rutgers.edu (X.Z.); inheon@rutgers.edu (I.H.L.); danmyers025@gmail.com (D.M.); borgiaja@rutgers.edu (J.A.H.); dayuang@pharmacy.rutgers.edu (D.G.); zoltan@pharmacy.rutgers.edu (Z.S.); 3 Department of Pharmacology and Toxicology, University of Utah, Salt Lake City, UT 84132, USA; avenosa1985@gmail.com; 4 Department of Biomedical Engineering, Rutgers, The State University of New Jersey, Piscataway, NJ 08854, USA; 5 Department of Biological Engineering, Princeton University, Princeton, NJ 08540, USA; prudhomm@princeton.edu; 6 Rutgers University CounterACT Research Center of Excellence, Piscataway, NJ 08854, USA; jl1450@eohsi.rutgers.edu; 7 Department of Pharmacology and Toxicology, Ernest Mario School of Pharmacy, Rutgers, The State University of New Jersey, Piscataway, NJ 08854, USA; laskin@eohsi.rutgers.edu

**Keywords:** M1 and M2 macrophages, drug delivery, mannose receptor, reprogramming macrophage polarization, HIV-1, tumor associated macrophages

## Abstract

Persistent activation of macrophages (MP)s into a proinflammatory M1 or anti-inflammatory M2 phenotype plays a role in several pathological conditions, including autoimmune diseases, fibrosis, infections, atherosclerosis and tumor development. The mannose receptor (MR, CD206), expressed at low levels on resting MPs and absent on M1 MPs, is highly expressed on M2 MPs, making it a potential target and drug delivery portal. Recently, we developed a novel, highly selective MR targeting ligand (MRTL), consisting of two mannose molecules separated by a monodisperse 12 unit poly(ethylene glycol) linker, to enhance the cellular uptake of polymeric nanocarriers. The feasibility of using the MRTL ligand for selectively targeting M2 MPs for intracellular delivery of nanoparticles (NPs) was investigated. Rat peritoneal MPs were differentiated into an M1 or M2 phenotype using IFN-γ and IL-4/IL-13, respectively. Expression of the M1 marker, inducible nitric oxide synthase (iNOS), and the M2 markers arginase (Arg)-1 and MR (at both the mRNA and protein levels) confirmed MP phenotypic activation. Resting, M1 and M2 MPs were treated with fluorescein isothiocyanate (FITC)-labeled MRTL or NPs displaying FITC-labeled MRTL at two surface densities (1 and 10%) and examined by confocal microscopy. Intracellular fluorescence was also quantified. Uptake of the MRTL was 2.4- and 11.8-fold higher in M2 MPs when compared to resting or M1 MPs, respectively, consistent with marker expression levels. Mannan, a competitive inhibitor of the MR, abrogated MRTL uptake. MRTL also co-localized with a fluid-phase endocytosis marker, further suggesting that uptake was mediated by MR-mediated endocytosis. Intracellular NP fluorescence was confirmed by flow cytometry and by confocal microscopy. MRTL-NPs accumulated intracellularly with no significant cell surface binding, suggesting efficient translocation. NPs displaying a low surface density (1%) of the MRTL exhibited significantly higher (2.3-fold) uptake into M2 MPs, relative to resting and M1 MPs. The 10% MRTL-NPs displayed greater uptake by M2 MPs when compared to resting and M1 MPs, but less uptake than 1% MRTL-NPs into M2 MPs. Control FITC-labeled plain NPs did not exhibit selective MP uptake. These studies demonstrate that M2 MPs are selectively targeted by NPs displaying a novel bivalent ligand that utilizes the MR as a target/portal for cell entry. This study also establishes the feasibility of the approach allowing for further investigation in vivo.

## 1. Introduction

In response to injury or infection, bone marrow-derived monocytes accumulate in tissues where they differentiate into distinct macrophage (MP) subsets, broadly defined as M1 proinflamatory/cytotoxic and M2 anti-inflammatory/wound repair, depending on environmental signals they encounter and intracellular regulatory pathways that are activated [1]. Whereas M1 MPs typically appear at sites of inflammation early after exposure to a noxious stimuli and release mediators that promote inflammation and induce cytotoxicity, M2 MPs accumulate later, releasing mediators that down-regulate the activity of M1 MPs and induce wound repair. It is thought that the outcome of inflammatory responses depends on the relative activity of these two MP subpopulations. In this context, overactivation of either M1 or M2 MPs leads to injury and disease pathogenesis. Thus, the very same macrophage-derived mediators released in controlled amounts by M1 MPs to destroy injurious materials and pathogens and by M2 MPs to initiate wound repair, when released in excess, can exacerbate tissue injury and/or induce chronic diseases such as fibrosis and cancer. Abnormal MP activation has been implicated in a variety of pathological states including autoimmune diseases [2], bacterial infection [3], atherosclerosis [4], obesity and metabolic syndrome [5], tumor progression [6], and HIV infection [7,8].

M1 and M2 MPs have been largely been identified based on their expression of specific cell surface and intracellular proteins and their functional responses [9,10]. However, these cells are highly plastic, which allows them to respond to a changing environment and modify their phenotype and activity [11]. In this regard, increasing evidence suggests that MP activation is a highly active process; hence, cells that initially promote inflammatory and cytotoxic reactions can readily undergo phenotypic switching, subsequently participating in the resolution of inflammation and injury [12,13,14]. These findings underscore the dynamic nature of the MP activation process and the notion that within any inflamed tissue, mixed phenotype MPs co-exist with M1 and M2 MPs, their specific function depending on the balance of activating and inhibiting activities and the tissue microenvironment [15].

Macrophage reprogramming is receiving increasing attention as a potential therapeutic approach to modifying disease progression [16]. In the case of pathological conditions associated with overactivated M1 MPs, new approaches aimed at suppressing or reversing the M1 phenotype are emerging [17,18,19,20,21]. In contrast, while it is well recognized that M2 MPs play a key role in the development of fibrotic diseases [22,23] and tumors [24], and that reprogramming of these cells would likely result in reversal of these pathological conditions [22,23,25,26,27,28,29,30,31], this has yet to be accomplished. This is due in part to the paucity of specific M2 MP proteins that are targetable [18,32]. One potential target that we have focused on is the mannose receptor (MR), which is highly expressed on M2, but not M1 MPs. The MR is a 175 kDa integral membrane protein. It recognizes and internalizes mannose, fucose and N-acetylglucosamine on the termini of polycarbohydrates [33,34,35]. This is accomplished via its C-type lectin-like domains (CTLDs)—two of which have appreciable binding affinity for mannose. MR–microbial mannose interactions are multivalent, resulting in an enhanced total binding affinity (i.e., avidity), since the natural ligands consist of repeated polycarbohydrates on many bacteria and virus surfaces with terminal mannose units. The MR also mediates cellular internalization of many non-opsinized natural ligands by endocytosis [36,37]. In addition to investigations into the biochemistry and molecular pathology of the MR, significant efforts have been made in recent years to explore the feasibility of MR-mediated drug delivery [38].

Previously, we constructed a novel MR-targeting, mannosylated nanocarrier and optimized its configuration in terms of carrier polymer size, mannose valency and the distance between individual mannose moieties [39]. In the present studies, the feasibility of using the optimized ligand, MRTL, and NPs displaying a low surface density of the ligand to specifically target M2 MPs was assessed. Significantly, we observed close to 12-fold and 2.3-fold greater uptake of MRTL and NPs displaying a low density of MRTL, respectively, into M2 MPs relative to M1 MPs, demonstrating high targeting specificity. These findings provide proof of principle that MRTL can facilitate cellular translocation of macromolecules such as nanoparticles. These findings also suggest that the MR can be utilized as a portal for intracellular NP delivery to MP subpopulations exhibiting the M2 phenotype. Finally, these studies lay a solid foundation for comprehensive in vivo studies.

## 2. Materials and Methods

All experimental procedures were part of protocol PROTO999900310/01-014 (approved 27 Mar 2018), approved by the Rutgers Institutional Animal Care and Use Committee, and were conducted according to the National Institutes of Health’s Guide for the Care and Use of Laboratory Animals.

### 2.1. Materials

Sieber amide resin was purchased from Anaspec (Fremont, CA, USA), Fmoc-L-serine-OH and Fmoc-L-norleucine(ε-N_3_)-OH from Chem-Impex (Wood Dale, IL, USA), α-D-mannose pentaacetate, boron trifluoride diethyl etherate, 4′,6-diamidino-2-phenylindole (DAPI), N,N-diisopropylethylamine (DIPEA) and anhydrous sodium methoxide in methanol from Sigma Aldrich (St. Louis, MO, USA), trifluoroacetic acid from Fisher Chemical (St. Louis, MO, USA) and Fmoc-dPEG_x_-acids ( 4 and 12) from Quanta Biodesign (Powell, OH). Polycaprolactone (PCL)-polyethylene glycol (PEG) diblock copolymers (PCL_6kDa_-PEG_5kDa_-OMe, PCL_6.5kDa_-PEG_5kDa_-NH_2_) were provided by Polymer Source, Inc. (Dorval, QC, Canada). Dibenzocyclooctyl reagent (DBCO-PEG_5_-NHS) for copper-free click chemistry was acquired from Click Chemistry Tools (Scottsdale, AZ, USA). Recombinant rat interferon gamma was purchased from EMD Millipore (Billerica, MA, USA), and recombinant rat IL-4, IL-10 and IL-13 from R&D Systems (Minneapolis, MN, USA). Antibodies (Abs) used were: Arg-1 and iNOS from BD Bioscience (San Jose, CA, USA), Cox-2, Mac-3 and MR from Abcam (Cambridge, MA, USA), Gal-3 from R&D Systems (Minneapolis, MN, USA) and actin from Santa Cruz Biotechnology (Dallas, TX, USA). HRP-conjugated anti-goat IgG was purchased from Santa Cruz Biotechnology (Dallas, TX, USA), HRP-conjugated anti-rabbit IgG and anti-mouse-IgG from GE Healthcare BioSciences (Piscataway, NJ, USA), and HRP-conjugated anti-chicken IgY from Thermo Fisher Scientific (Waltham, MA, USA).

### 2.2. Quantitative Real-Time PCR

Total RNA was extracted from MPs using the RNeasy Mini Kit (Qiagen, Valencia, CA), and the RNA was reverse-transcribed using the High Capacity cDNA Reverse Transcription Kit (Applied Biosystems, Waltham, MA, USA) according to the manufacturer’s protocol. Standard curves were generated using serial dilutions from pooled randomly selected cDNA samples. Samples from each treatment were analyzed, and results were presented relative to glyceraldehyde-3-phosphate dehydrogenase (GADPH) mRNA expression. Real-time PCR was performed using SYBR Green PCR Master Mix (Applied Biosystems, Waltham, MA, USA) on a 7900HT Thermocycler, using 96-well optical reaction plates according to the manufacturer’s protocol. All PCR primer sequences were generated using Primer Express 3.0 (Applied Biosystems, Waltham, MA, USA), and primers were synthesized by Integrated DNA Technologies (Coralville, IA, USA). A minimum of three samples was analyzed for each experimental group, and all samples were run in duplicate. Primer sequences were as follows: inducible nitric oxide synthase (iNOS): (F) TGGTGAAAGCGGTGT TCTTTG, (R) ACGCGGGAAGCCATGA; Arginase-1 (Arg-1): (F) CCAAGCCAAAGCCCA TAGAG (R) TCCTCGAGGCTGTCCCTTAG; and GAPDH: (F) CCTGGAGAAACC TGCGAAGTAT, (R) CTCGGCCGCCTGCTT.

### 2.3. Western Blotting

MPs were lysed in buffer (20 mM HEPES, 150 mM NaCl, 10% glycerol, 1% Triton X-100, 1.5 mM MgCl_2_, 1 mM diethylene triamine pentacetic acid, 1 mM phenylmethylsulfonylenediamine, 10 mM sodium pyrophosphate, 50 mM sodium fluoride, 2 mM sodium orthovanadate) and a protease inhibitor cocktail from Sigma (St. Louis, MO, USA). Protein concentrations in the lysates were determined using a BCA Protein Kit from Pierce (Rockford, IL, USA), with bovine serum albumin as the standard. Proteins in the lysates were separated on 4–12% gradient polyacrylamide gels from Invitrogen (Carlsbad, CA, USA), transferred to nitrocellulose membranes, blocked at room temperature with a blocking buffer (5% nonfat dry milk, 10 mM Tris-base, 200 nM sodium chloride and 0.1% Tween 20) for 60 min, and then incubated overnight at 4 °C with the mouse monoclonal iNOS antibody (1:500), polyclonal rabbit anti-MR antibody (1:1000), or monoclonal mouse anti-Arg-1 antibody (1:1000, BD). This was followed by incubation with goat anti-mouse horseradish peroxidase (HRP), donkey anti-goat IgG-HRP, or anti-rabbit IgG HRP (1:10,000) for 1 h at room temperature. Target proteins were detected using ECL Plus (GE Healthcare, Piscataway, NJ, USA).

### 2.4. Preparation of Bivalent Mannose Receptor Targeting Ligand (MRTL)

**Figure 1:**
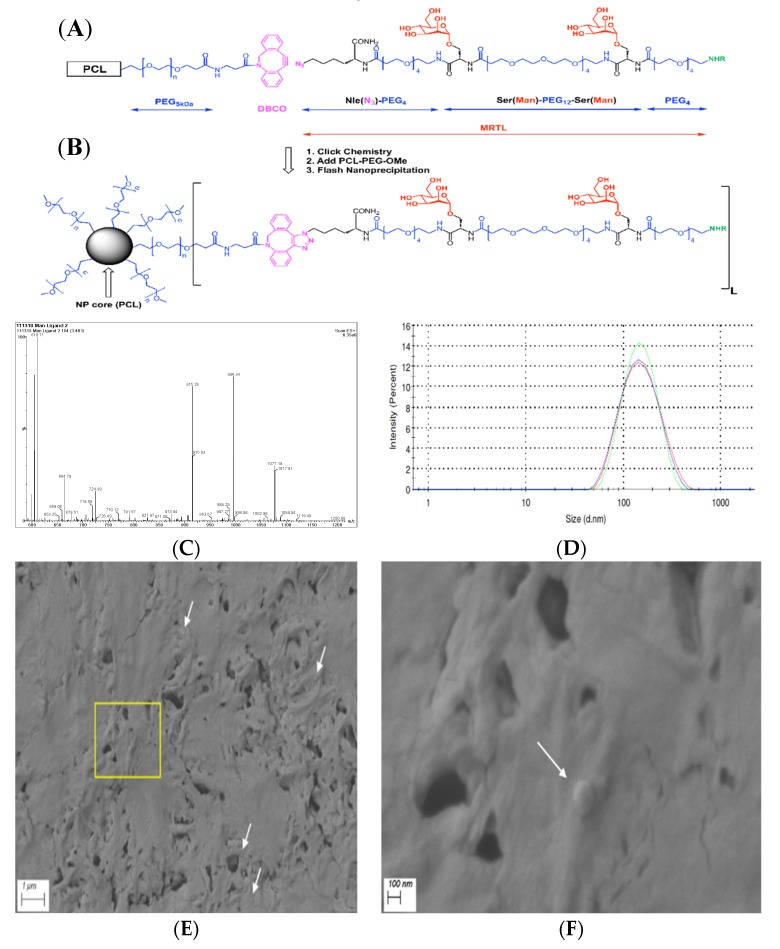
Chemical structures of polymer and mannose receptor targeting ligand (MRTL), conjugation chemistry, schematic representation and characterization of nanoparticles (NPs). (**A**) Polycaprolactone (PCL)-polyethylene glycol (PEG)-dibenzocyclooctyl (DBCO) polymer and unconjugated MRTL; (**B**) polymer conjugated ligand as displayed on NP surfaces; color code: mannose units are in red, PEGs (including 5 kDa polymers and monodiscrete 4 and 12 unit long spacers) are in blue, click chemistry precursors (DBCO and azido units) and the product are in purple, and fluorescent labeling at the N-termini of the ligands are in green (NHR represents fluorescein isothiocyanate (FITC) reacted with the N-termini forming stable thiourea bonds); (**C**) ESI-MS spectrum of the main HPLC peak of MRTL at 3.40 min; consisting of characteristic ions, e.g., m/z = 1077.19 and 718.58 for [M+2H]/2 and [M+3H]/3. The calculated m/z values are 1076.78 and 718.32, respectively; (**D**) particle size distribution (in triplicate) of the low-surface-density (1%) MRTL-NPs (MRTL_1%_-NPs), diameter = 133.7 ± 2.3 nm; and PDI = 0.150 ± 0.023. Control (plain) NPs and high-surface-density (10%) MRTL-NPs (MRTL_10%_-NPs) had similar sizes and distributions (data not shown); and SEM micrographs of plain PCL-PEG NPs at (**E**) 20,000× and (**F**) 100,000× magnifications. White arrows highlight some NPs. (**F**) depicts the zoomed-in image of the yellow box in (**E**).

Fmoc-L-Ser[α-D-Man(OAc)_4_]-OH building block for solid supported peptide synthesis was synthesized using the procedure of Kragol et al. [40] and utilized as described in our preceding publication on ligand optimization [39]. The MRTL [fluorescein isothiocyanate (FITC)-PEG_4_-L-Ser(α-D-Man)-PEG_12_-L-Ser(α-D-Man)-PEG_4_-L-Nle(ε-N_3_)-CONH_2_, shown in Figure 1A] for NP conjugation was synthesized on a solid support, utilizing a Nautilus 2400 synthesizer. NovaSynTG Sieber resin was chosen for the synthesis due to its PEG-grafted, polystyrene-core structure, which facilitates the assembly of difficult peptide sequences. Standard analytical procedures—Kaiser test, test cleavages followed by analytical high-performance liquid chromatography-mass spectrometry (HPLC-MS)—were applied. For analytical HPLC-MS, an Eclipse Plus C_18_ column (4.6 × 50 mm, particle size 3.5 µm, Agilent Technologies Inc.) was used. After cleavage and prior to final purification, the acetyl protecting groups of the mannose moieties were removed by sodium methoxide in methanol, as described in our previous publication [39]. Semi-preparative reversed phase (RP)-HPLC (C_18_ column, 22 × 250 mm, Vydac; particle size 10 µm, pore size 12 nm) was used to purify the product, followed by HPLC-MS fraction analysis and lyophilization.

### 2.5. Preparation and Characterization of Plain Nanoparticles (NPs) and NPs Displaying MRTL

The copy number of MRTL displayed on the NP surface is directly related to the initial ratio of mannose-polymer conjugate to unmodified block copolymer, as shown in Figure 1A,B. Copper-free click chemistry was utilized for the MRTL-block co-polymer conjugation. The two-step procedure was described in our previous publication [41]. Briefly, dibenzocyclooctyne-PEG_5_-carboxylic acid N-hydroxysuccinimide ester (DBCO-PEG_5_-NHS), a click chemistry reagent, was conjugated to an amine-functionalized copolymer (PCL-PEG-NH_2_) in the presence of 10 eq. of N,N-diisopropylethylamine. The FITC-labeled mannose ligand (carrying the azido functional group) was then conjugated to the DBCO-conjugated polymer in DMF, overnight with light stirring. The resulting mannose-polymer conjugate was mixed with unmodified block copolymer (PCL-PEG-OMe) as shown in Figure 1A,B (step 2), and the resulting polymer mixture was used to make NPs by flash nanoprecipitation (FNP) [42]. To allow for adequate NP detection, free FITC was conjugated to PCL-PEG-NH_2_, and the resulting FITC-polymer conjugates were added to the polymer mixture at a ratio of at least 7 wt%. The polymer mixtures for the three different NP formulations were as follows: 1) plain NPs consisting of 13.95 mg PCL-PEG, 1.05 mg FITC-polymer, 2) low-surface-density (1%) MRTL-NPs (MRTL_1%_-NPs) consisting of 13.95 mg PCL-PEG, 0.9 mg FITC-conjugated polymer, 0.15 mg MRTL-conjugated polymer, and 3) high-surface-density (10%) MRTL-NPs (MRTL_10%_-NPs) consisting of 13.5 mg PCL-PEG, 1.5 mg MRTL-conjugated polymer. For FNP, the copolymer mixture was dissolved in THF at 15 mg/mL. Vitamin E (VitE) was added to the mixture as a hydrophobic core material, at a 1:1 mass to copolymer ratio. The polymer and VitE solution was mixed rapidly with an equivalent volume of DI water, by injecting into a dual-inlet vortex mixer. The outlet was collected DI water. The resulting NP suspension was vortexed for a few seconds immediately after FNP. The NP suspension was then dialyzed against 4 L of DI water overnight, to remove the remaining organic solvent. DI water was replaced at least twice during dialysis. The dialyzed NPs were characterized by dynamic light scattering (DLS) using a Zetasizer Nano-ZS90 (Malvern Instruments Ltd., Malvern, UK). The sizing measurements were performed in triplicates at a measurement angle of 90° in plastic cuvettes (dimensions: 12.5 × 12.5 × 45 mm) and analysed by Zetasizer Software (version 7.12), resulting in acceptable polydispersity indices (PDIs). Representative samples of NPs were visualized by a Sigma field emission scanning electron microscope (FESEM) (ZEISS Microscopy, Jena, Germany). Samples were prepared by drop casting the NP suspension on a carbon tape attached to a sample holder, followed by sputter coating with a 15-nm-thick layer of gold prior to observation. Images were obtained at the acceleration voltage of 3 kV.

### 2.6. Rat Peritoneal MP Isolation and Phenotypic Activation

Male Sprague–Dawley rats (200–225 g, 8–9 weeks) were purchased from Harlan Laboratories (Frederick, MA, USA). The rats were allowed to acclimate to the animal facility for a minimum of 5 days prior to use. The animals were euthanized by CO_2_ asphyxiation. Twenty milliliters of ice-cold PM1 perfusion medium (0.5 mM EGTA, pH 7.2, Ca^2+^ and Mg^2+^ free) was injected into the peritoneal cavity three times. After each injection, the peritoneum was massaged for 30 s, and the solution containing peritoneal MPs was withdrawn. MPs were washed three times with PBS (300× *g*/4 °C, 8 min), suspended in Dulbecco’s modified Eagle medium (DMEM) containing 10% FBS and then inoculated into 24-well plates or chambered coverglasses. After 2 h at 37 °C, adherent MPs were rinsed three times, refed with DMEM containing 10% FBS. After overnight incubation at 37 °C, the cells were rinsed and incubated for an additional 6, 24, 48, and 72 h in DMEM containing 1% FBS plus and minus IFN-γ (20 ng/mL) or IL-4 (10 ng/mL) + IL-13 (10 ng/mL) for M1 or M2 activation, respectively.

### 2.7. Macrophage Uptake

Activated MPs were incubated for 1 h with phenol red-free DMEM containing 1% FBS, 120 nM of FITC-labeled MRTL or non-mannosylated control, 5 μM of the general fluid enocytosis marker rhodamine-dextran (MW 10,000) and 5 μg/mL of the nuclear dye DAPI. In some experiments, the macrophgaes were preincubated for 40 min with medium containing 3 mg/mL mannan (a MR ligand serving as a competitive inhibitor). Cells were then washed twice with cold phosphate buffered saline (PBS), twice with acid wash solution (0.5 M sodium chloride and 0.2 M acetic acid, pH 2.5), and twice with cold PBS. The cells were analyzed immediately for intracellular fluorescence (see below). Cellular uptake of NPs displaying a low (1%) or high (10%) density of the MRTL, or control NPs, was conducted similarly with the following modification: isolated MPs were plated on 12-well plates and activated as described above. The cells were then incubated with NPs and washed three times with Hank’s buffered saline solution (HBSS). The cells were then detached (using a cell scraper) in 0.5 mL of 10% formalin solution per well. Resuspended cells were analyzed by flow cytometry or plated onto coverglass chambers pre-coated with poly-D-lysine for confocal microscopy, as described below.

### 2.8. Confocal Microscopy

Activated MPs were incubated for 1 h at 37 °C with medium containing MRTL or ligand control and washed as described above. Cells were analyzed on a Leica SP5 confocal microscope, using the XYZ mode. Stacks of images—0.41 μm in thickness—were collected along the Z-axis. An image of a middle section of each Z stack was used to quantify uptake, using Leica LAS AF Lite analytical software. Ten randomly chosen cells in five fields from each MP group were determined in arbitrary units of the software, and data expressed relative to M1 cells. For uptake of NPs, a similar procedure was performed. A middle section of a Z stack of cells treated with each type of NP was presented to confirm that the fluorescence of the NPs was located inside the cells; no quantification of intracellular fluorescence intensity was performed since flow cytometry yielded the same information.

### 2.9. Flow Cytometry

Activated MPs in 12-well plates were incubated with medium containing each type of NP at 3 nM and DAPI at 5 μg/mL, in a CO_2_ incubator for 3 h. Attached cells were washed twice with HBSS, detached with a cell scraper, in the presence of 0.5 mL of 10% formalin solution, and analyzed by flow cytometry using a Beckman Coulter Gallios flow cytometer. Since the absolute fluorescence intensities of different NPs differed from each other, uptake levels were measured in the percentage of cells having fluorescence intensities above that of untreated cells.

## 3. Results

### 3.1. Synthesis of the Optimized Mannosylated Ligand (MRTL)

In our previous studies, the configuration of a MR-targeting mannosylated polymeric nanocarrier was optimized in terms of PEG polymer size, mannose unit copy number and the distance between mannose moieties on the nanocarrier [39]. In this study, the optimized parameters (two mannose units, PEG_12_ spacer) for the bivalent MRTL were used (i.e., two mannose moieties linked together by a short monodisperse PEG linker). MRTL was synthesized by standard solid-supported “Fmoc”-peptide chemistry and purified by reverse-phase HPLC using a C_18_ column. The fractions were analyzed by HPLC-MS and pooled. The lyophilized product showed very high purity (97.9% by HPLC; not shown), as well as conforming ions by electrospray ionization (ESI)-MS (Figure 1C). Based on these ions, the measured exact mass was 2152.54 Da, matching the calculated monoisotopic exact mass of 2151.95 Da. The structure of the MRTL with two copies of mannose is shown in Figure 1A,B.

### 3.2. Fabrication and Characterization of MRTL-Nanoparticles

Flash nanoprecipitation yielded stable NPs (Plain, MRTL_1%_-NPs and MRTL_10%_-NPs) after three rounds of dialysis [42]. DLS measurements revealed a reproducible, narrow particle size distribution (as shown in Figure 1D) for MRTL_1%_-NPs. MRTL_1%_-NPs were characterized by a diameter of 133.7 ± 2.250 nm and a PDI of 0.150 ± 0.023. MRTL_10%_-NPs had a similar size distribution, with a diameter of 132.6 ± 0.6429 nm and a PDI of 0.209 ± 0.005. Fluorescence spectroscopy was used to confirm the presence of total FITC (both conjugated to PCL-PEG and conjugated to MRTL) on NP surfaces. The surface morphology of the NPs was observed with a FESEM. As shown in Figure 1E,F, the particles were spherical and had a diameter of 100–150 nm, consistent with DLS measurements. Sporadic NP spheres, rather than clusters, might be due to the high concentration of the NP suspension that was drop casted on the carbon tape during sample preparation. To confirm the morphology, the freeze-dried powder of the same NPs was also observed with a FESEM and showed clusters of spheres covered by cryoprotectants (images not shown).

### 3.3. Characterization of Activated Rat Peritoneal MPs

**Figure 2:**
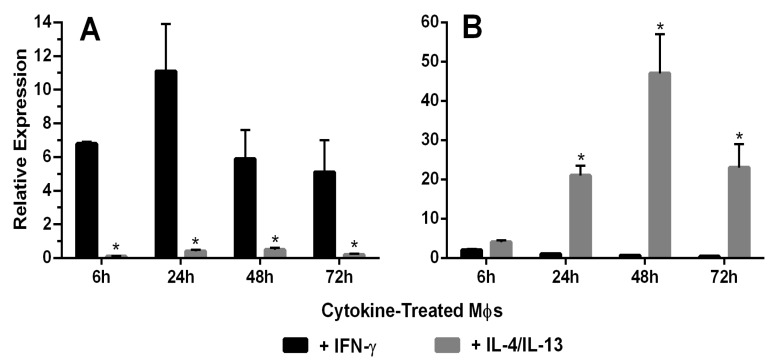
(**A**) Macrophage expression of the M1 marker iNOS. iNOS mRNA expression in rat peritoneal macrophages (MΦ) treated with IFN-γ or IL-4/IL-13 for 6–72 h was characterized using qRT-PCR (*n* = 5). The 65-, 28-, 13- and 19-fold differences between IFN-γ and IL-4/IL-13-treated macrophages were observed at 6, 24, 48 and 72 h, respectively (*: *t* test, *p* < 0.05). (**B**) Macrophage expression of the M2 marker Arg-1. Arg-1 mRNA expression by rat peritoneal macrophages treated with IFN-γ or IL-4/IL-13 for 6–72 h was characterized using qRT-PCR (*n* = 5). The 32-, 135- and 265-fold differences were observed between IFN-γ and IL-4/IL-13-treated macrophages at 6, 24, 48 and 72 h, respectively (*: *t* test, *p* < 0.05).

**Figure 3:**
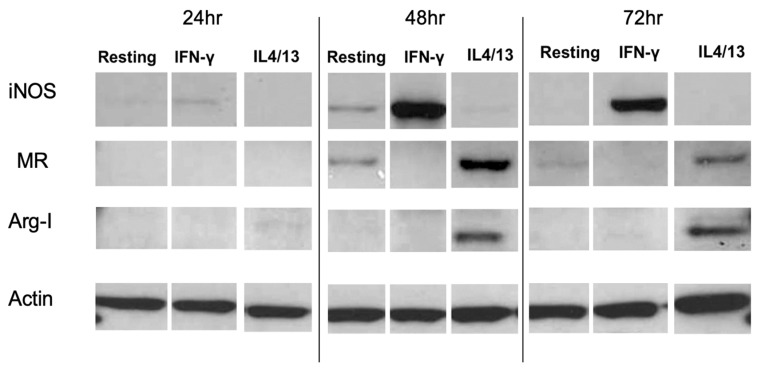
Time course of protein expression levels of the M1 marker iNOS, the M2 markers Arg-1 and MR, and the housekeeping protein actin in activated MPs. Western blot was performed using cell lysates from activated MPs. IFN-γ-treated MPs expressed high levels of iNOS protein at 48 and 72 h, while untreated resting and IL-4/IL-13-treated MPs expressed no or low levels of iNOS protein at 48 and 72 h. Conversely, IL-4/IL-13-treated MPs expressed high MR and Arg-1 levels at 48 and 72 h, while the IFN-γ-treated MPs expressed no or low levels of MR and Arg-1 proteins. The result shown represents one of the independent experiments.

Figure 2A shows mRNA expression of the M1 marker iNOS after IFN-γ or IL-4/IL-13 treatment for 6, 24, 48 and 72 h. Significantly increased iNOS mRNA expression was observed at all time points in IFN-γ-treated MPs, with the highest expression occurring at 24 h. Figure 2B shows mRNA expression of the M2 marker Arg-1 after IFN-γ or IL-4/IL-13 treatment for 6, 24, 48 and 72 h. Significantly increased Arg-1 mRNA expression was observed 24–72 h after treatment, with the highest expression occurring at 48 h. Figure 3 shows the time course of protein expression of iNOS and Arg-1 in activated macrophages. iNOS expression peaked at 48 h in MPs treated with the M1 inducer IFN-γ, consistent with the qRT-PCR results. MR expression and Arg-1 expression were evident in the IL-4/IL-13-treated MPs, with peak levels occurring at 48 h. The characterization of marker expression at both the mRNA and the protein levels verified the M1 and M2 MP phenotypes.

### 3.4. MR-Mediated MRTL Uptake into Activated MPs

**Figure 4:**
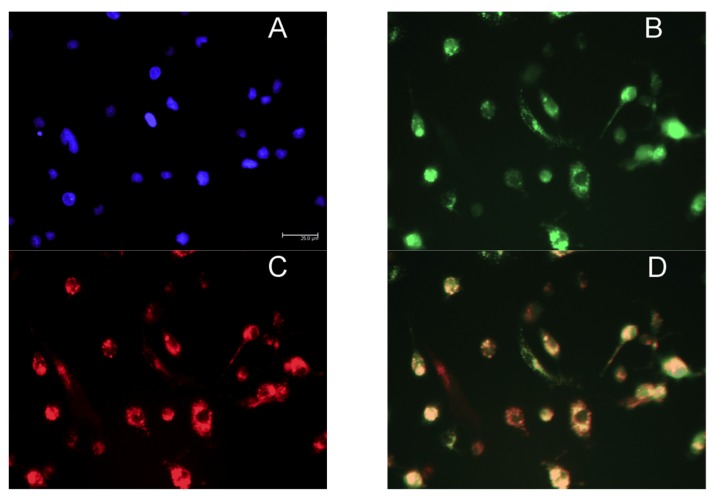
Fluorescence microscopy images showing co-localization of FITC-labeled mannosylated ligand with the fluid endocytosis marker rhodamine-dextran. Rat M2 MPs were incubated with culture medium containing the nuclear dye DAPI, 120 nM FITC-labeled MRTL and 5 µM of the general fluid endocytosis marker rhodamine-dextran, for 1 h. Cells were washed with PBS buffer and acid wash solution and analyzed by fluorescent microscopy using a light filter for DAPI ((**A**) blue), FITC ((**B**) green) and rhodamine-dextran ((**C**) red). The three-color merged image (**D**) shows that the green and red fluorescence are largely co-localized, resulting in an orange color. This suggests that the free MRTL is internalized and situated mainly in endocytosed vesicles.

**Figure 5:**
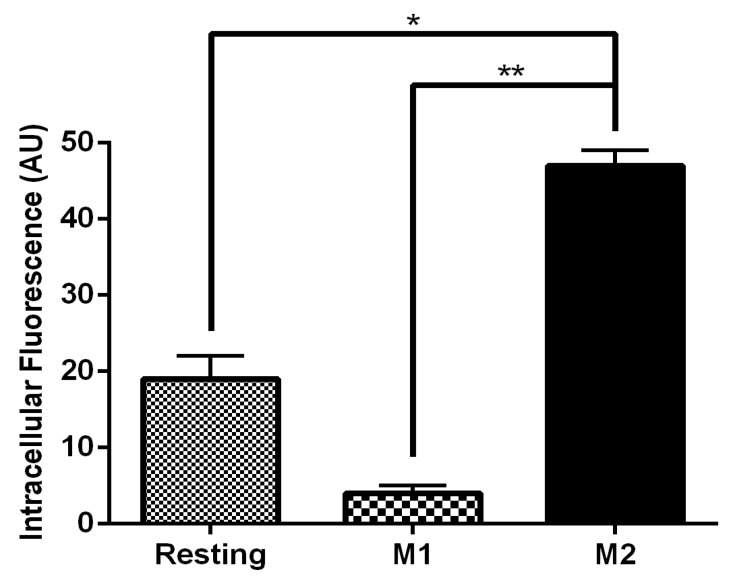
Uptake of FITC-labeled MRTL into M1 and M2 MPs. Free MRTL (120 nM) was incubated with macrophages for 1 hour at 37 °C. Cells were washed with PBS and acid wash solution and analyzed by confocal microscopy. Quantification of the uptake was performed with the confocal image analytical software. Compared to M1 MPs, uptake into resting MPs and M2 MPs was 2.4- and 11.8-fold greater, respectively (AU: arbitrary units of fluorescence of the confocal analytical software; *: *t* test *p* < 0.05; **: *t* test *p* < 0.01).

**Figure 6:**
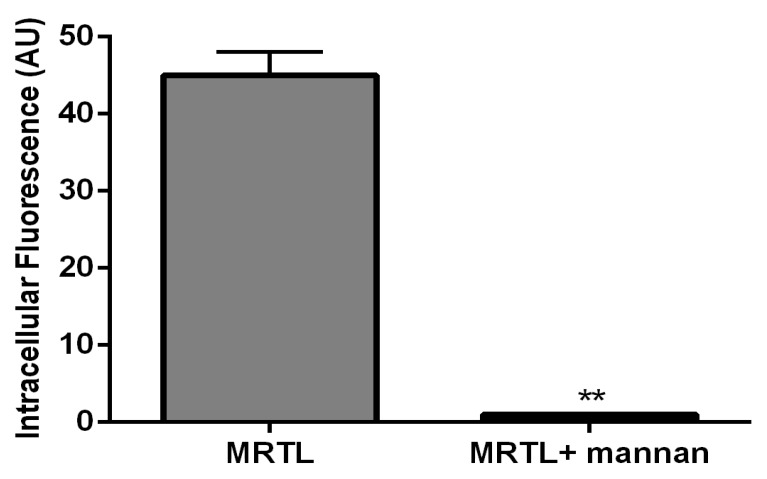
A natural MR ligand, mannan, inhibited the uptake of the FITC-labeled bivalent MRTL by rat M2 MPs. M2 MPs were incubated with 3 mg/ml of mannan for 40 minutes at 37 °C, followed by MRTL to a final concentration of 120 nM and further incubation for 1 hour at 37 °C. The attached cells were washed twice with cold PBS, twice with acid wash solution (0.5 M sodium chloride and 0.2 M acetic acid, pH 2.5), and twice with cold PBS. Intracellular fluorescence assessed by confocal microscopy was quantified using analytical software. The presence of mannan resulted in a 98.5-fold reduction in intracellular fluorescence (**: *t* test, *p* < 0.01).

Figure 4 shows fluorescent images of M2 MPs treated with free MRTL, in which the green FITC-labeled mannosylated ligand co-localizes with the red general (fluid phase) endocytosis marker rhodamine-dextran. Not all of the red punctate fluorescence is co-localized with the green punctate fluorescence, suggesting that uptake occurred through endocytosis by a specific receptor-mediated pathway. The greatest intracellular green fluorescence was observed in M2 MPs, followed by resting and then M1 MPs. These findings are in agreement with the order of expression levels of M2 markers. The intracellular green fluorescence assumed a punctate appearance in both resting and M2 MPs (confocal images not shown). Figure 5 shows the quantification of intracellular green fluorescence, which indicated that compared to the fluorescence of the M1-activated MPs (1×), M2-activated MPs exhibited 11.8-fold greater fluorescence and the non-activated resting cells exhibited 2.4-fold greater fluorescence. The correlation between MR expression and uptake strongly suggests that MRTL uptake was mediated by the MR, since M2 MPs exhibited the highest MR protein expression, resting cells had low expression and the M1 MPs had barely detectable MR protein expression. To confirm this, a competitive inhibition uptake experiment was carried out. Namely, MRTL uptake was investigated in the absence and presence of a non-toxic concentration of mannan, a mannose polymer known to specifically and competitively bind to the MR. The results, shown in Figure 6, indicate that mannan completely abolished MRTL uptake into M2 MPs, suggesting that the MR exclusively mediates ligand uptake.

### 3.5. MR-Mediated Uptake of MRTL-NPs by Activated MPs

**Figure 7:**
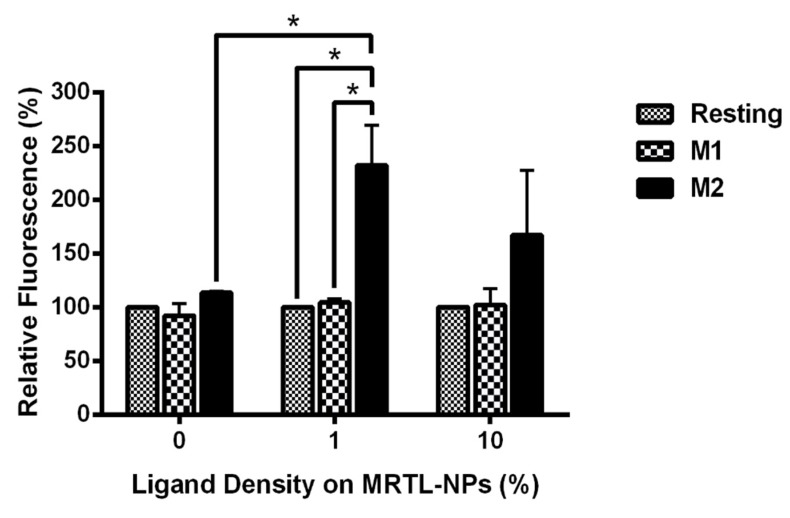
Uptake of FITC-labeled MRTL-NPs with 1% or 10% of the total copolymer termini displaying the mannosylated ligand. Data are the average of two independent rat experiments, each using macrophages pooled from at least two rats. M1 and M2 activated macrophages were incubated with DAPI and either plain (0%) NPs, MRTL_1%_-NPs or MRTL_10%_-NPs. After incubation, the cells were washed and scraped from the dishes and fixed in 10% formalin in PBS. Cell-associated FITC fluorescence in the fixed cells was then assessed by flow cytometry. The values are expressed relative to that of resting macrophages (i.e., the value of resting macrophages is 100%; *: *t* test *p* < 0.05).

**Figure 8:**
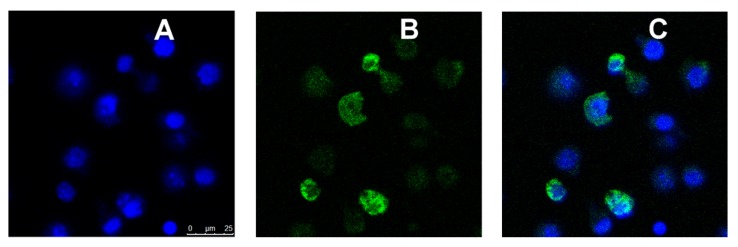
Confocal microscopic images of M2 MPs treated with FITC-labeled MRTL_1%_-NPs. Activated M2 MPs were inoculated into coverglass chambers and analyzed by confocal microscopy, conducted in the XYZ mode. A middle section of 0.5 μm thickness in a Z stack is shown. (**A**) DAPI (blue) fluorescence image. (**B**) FITC (green) fluorescence image. (**C**) Blue and green merged images. The green NPs are within the cells, with no significant cell surface binding.

We next determined whether NPs displaying the MRTL can specifically target and be internalized by M2 MPs. FITC-labeled plain NPs, displaying no ligand (control NPs), and FITC-labeled MRTL_1%_-NPs and MRTL_10%_-NPs were fabricated. The percentage represents the ratio of mannosylated copolymer termini to total copolymer termini on the NP surface. M1 and M2 activated and resting MPs were treated with medium containing DAPI and each of the three NPs at 3 nM, followed and then analyzed by flow cytometry. As expected, plain NPs did not show a significant difference in fluorescence between the different MP populations (Figure 7). The MRTL_1%_-NPs uptake by M2 MPs was statistically different from resting and M1 MPs, with a 2.3-fold higher cellular FITC fluorescence in M2 MPs versus resting and M1 MPs. No differences were observed between resting and M1 MPs. The MRTL_10%_-NPs poorly differentiated between different phenotypes, with only a 1.7-fold higher cellular FITC fluorescence in M2 versus resting or M1 MPs. As discussed below, the configuration of the NPs prepared by flash nanoprecipitation was not fully optimized in terms of displaying the mannose moieties of the MRTL, which resulted in the sub-par differentiation between M2 and the resting and M1 phenotypes as compared to the differentiation made by the free mannosylated ligand/MRTL. Confocal microscopy showed that the FITC fluorescence of low-surface-density MRTL-NPs in the middle section of the cells was located exclusively within the cells (Figure 8), suggesting that the difference between M2 and resting/M1 MPs represents a difference of intracellular NPs.

## 4. Discussion

While drug delivery to MPs has been explored for at least two decades, delivery and targeting to specific MP phenotypes is lacking [43,44,45,46,47]. Targeting MPs of a specific activated phenotype is an important goal, considering the significant body of literature demonstrating the role of MP phenotype in various diseases. Furthermore, it has been suggested by researchers in various fields that the reprogramming of activated MPs in pathological states may represent an effective therapeutic strategy. This effort requires delivery of regulatory molecules, often macromolecular proteins or nucleic acids.

Previously, we reported the design, fabrication and evaluation of polymeric mannose-bearing nanocarrier ligands, which were validated for MR binding and selectivity in resting (non-activated) murine MPs [39]. We found the optimal ligand configuration included two copies of mannose, a 12 kDa PEG carrier with 56 Å spacing between mannose units. The Ser(Man)-PEG_12_-Ser(Man) construct has numerous advantages, including a monodisperse structure and synthetic accessibility. The PEG spacer provides high flexibility for the backbone of the ligand, as well as suitable exposure for the mannose units. However, in order to facilitate the copper-free click chemistry for the MRTL-polymer conjugation, the cysteine residue at the C-terminus of the previous design was replaced by a ε-N_3_-norleucine (Figure 1A). The flexibility and steric accessibility (as depicted in Figure 1B) are especially important characteristics for optimal MR binding, due to its elongated, multicarbohydrate-recognizing domain architecture. The modular design allows for rapid optimization, while the late-stage polymer-ligand conjugation via copper-free click chemistry offers independent optimization of the MRTL and the polymer support.

In the present studies, rat MPs were induced to undergo phenotypic activation from a resting state to an M1 or M2 phenotype. Based on expression of the M1 marker iNOS and the M2 markers MR and Arg-1, it was demonstrated, at both the mRNA and protein levels, that the MPs were indeed phenotypically activated [48,49,50,51].

MRTL, the optimized polymeric mannose-bearing ligand from our previous study [39], was modified (Figure 1A) in order to support copper-free click chemistry and to facilitate precise attachment to the surface of NPs. The present studies demonstrate the MR-mediated uptake of the MRTL into M2 MPs was 2.4- and 11.8-fold higher than in resting and M1 MPs, respectively (Figure 5). The magnitude of difference among the three phenotypically distinct MPs correlated well with expression levels of M1 and M2 markers, thus demonstrating the feasibility of specifically targeting M2 macrophages. MRTL uptake into MPs appeared as punctate fluorescence and was co-localized with the fluid-phase endocytosis marker rhodamine dextran, consistent with MR-mediated uptake. The vast majority of MRTL was internalized with little surface binding, demonstrating efficient cell membrane translocation (Figure 4). MR-mediated uptake of MRTL was further supported by competitive inhibition studies that showed nearly complete (98.5-fold) (Figure 6) reduction of uptake in the presence of mannan, a natural MR ligand. This response is consistent with our previously published results in resting murine MPs, where it was shown that the presence of mannan reduced ligand uptake by >85% [39]. Song et al. [52], using a reversible addition-fragmentation chain transfer (RAFT)-based glycopolymer functionalized with either mannose or galactose, demonstrated dose-dependent uptake by means of endocytosis into bone marrow-derived macrophages (BMDMs). In M2 BMDM, uptake after one hour was ~3- and ~5-fold greater than in resting or M1 BMDM, respectively. The observed differences with the current MRTL results may be related to the size of the polymeric carrier, since the number averaged molecular weights of the RAFT glycopolymers ranged from 11,400 to 13,100 Da as compared to 2152 Da for MRTL. Interestingly, they did not observe a significant difference in uptake between the resting and M1 BMDM, even though resting macrophages express MR (Figure 3). This disparity could be due to differences in the cell lines that were used and/or activation protocols.

We next determined whether NPs displaying MRTL on the surface, which are much larger in size than MRTL or the RAFT glycopolymers previously reported [39,52], would be selectively and differentially taken up by resting, M1 and M2 MPs, and whether the surface display density of MRTL was a significant factor given the importance of clustering on uptake via the MR. MRTL-NPs were prepared at low (1%) and high (10%) surface density (i.e., percentage of mannosylated ends of total copolymer termini). Control/plain NPs were also prepared that contained copolymer termini displaying no mannose (0%). The results (Figure 7 and Figure 8) show that after one hour, the MRTL_1%_-NPs were internalized by M2 MPs 2.3-fold more efficiently than either resting or M1 MPs. MRTL_1%_-NP uptake into M2 MPs was significantly greater than in resting MPs, confirming that selectivity was maintained on the larger NP construct. The slight reduction in MRTL-NP uptake compared to the control into M2 MPs relative to MRTL alone (Figure 5) or the previously reported RAFT glycopolymers [52] suggests a molecular size dependence for uptake through the MR. There was no significant difference between MRTL_1%_-NP and MRTL_10%_-NP uptake into M2 MPs. The higher variability of MRTL_10%_-NP could be due to the fact that they were not fully optimized. It is known whether the mannose ligand density displayed on NPs affects the rate by which macrophages take up the NPs. For example, we have previously shown that mannose density displayed on NPs of the same type critically affected mannosylated NP uptake by murine J774E macrophage-like cells [53]. The surface mannose density on the NP also affects in vivo clearance, another property of mannose that must be considered and optimized [43]. It is also possible that the FITC moietes located at the tips of the non-mannosylated copolymer termini in the three types of NPs were subject to different molecular environments, resulting in varying degrees of FITC quench. This supposition implies that mannosylated copolymers in the low- and high-density mannosylated NPs are packed differently on the NP surfaces, leading to varying degrees of interactions between the mannosylated NPs and the MR. Finally, it is unlikely that the high-density surface PEG corona interfered with MR–mannose binding, as we have previously demonstrated that hydroxyl-terminated PEG NP coronas fully interacted with the MR at high mannose surface display densities, while methoxy-terminated PEG coronas passivated the NP surface and led to minimal interactions between the NPs and the MR [53]. Further optimization of the mannosylated NP is likely to improve the M2 targeting specificity and load efficiency.

## 5. Conclusions

This study shows, for the first time, that alternatively activated M2 MPs can be specifically targeted using a polymeric mannose targeting ligand displayed on a nanoscale macromolecular carrier. While low-surface-density MRTL-NPs demonstrated higher uptake than high-density MRTL-NPs into M2 MPs, the mechanism is not yet clear and requires further study. It may be due to the specific requirements of the mannose receptor, which is known to use clustering for cell uptake. These findings also validate the concept of delivery of: (1) regulatory effectors, to revert a harmful polarized macrophage population associated with a pathological condition or disease, or (2) traditional drugs, to the diseased tissues where these macrophages reside. Given the availability of a suitable cell surface marker for other types of activated macrophages, it is likely that these disparate macrophage subpopulations can also be targeted.
